# Comparison of Moducare versus Wait-and-See approach for histologically proven Low-grade Cervical Intraepithelial Neoplasia (CIN1) (MODUCIN1 TRIAL) – Study protocol

**DOI:** 10.1371/journal.pone.0353119

**Published:** 2026-07-13

**Authors:** Dimitrios Zouzoulas, Iliana Sofianou, Kimon Chatzistamatiou, Vasilis Theodoulidis, Grigoris Grimbizis, Dimitrios Tsolakidis

**Affiliations:** 1st Department of Obstetrics & Gynecology, Aristotle University of Thessaloniki, “Papageorgiou” Hospital, Thessaloniki, Greece; UFRN: Universidade Federal do Rio Grande do Norte, BRAZIL

## Abstract

Human Papillomavirus (HPV) is causally associated with cervical cancer and precancerous lesions (dysplasias) of the cervix. The treatment of choice for low-grade lesions is monitoring with no-treatment, because the majority of them will regress spontaneously. However, this wait-and-see approach can be assisted by nutritional supplements for immune support, like Moducare. The hypothesis of the trial is that Moducare can enhance the natural regression of CIN1. MODUCIN1 is a prospective, open-label, randomized trial where eligible patients will be randomized (1:1) to either the wait-and-see approach (control arm) or to the six months oral administration of Moducare capsules. The main inclusion criterion is newly histologically proven CIN1 and the main exclusion criterion is any previous cervical intraepithelial neoplasia with or without treatment or history of pelvic malignancy. The primary objective is to compare the regression rates of low-grade cervical intraepithelial neoplasia (CIN1) between the two groups: Wait-and-see approach and Moducare. The sample size is estimated at 182 eligible patients, while accrual is expected to last one year. The primary endpoint is expected to be reached after six months from last patient enrollment. The Trial Registration Number is NCT07379905.

## Introduction

Human papillomavirus (HPV) is causally associated with virtually all cases of cervical cancer and its precursor lesions under the term cervical intraepithelial neoplasia (CIN), representing one of the strongest causal relationships documented in cancer epidemiology [1]. HPV-induced cervical dysplasia arises predominantly within the transformation zone and is classified histologically into low-grade cervical intraepithelial neoplasia (CIN1) and high-grade cervical intraepithelial neoplasia (CIN2/CIN3) lesions according to the Bethesda classification system, with the latter warranting excisional treatment. CIN1 lesions demonstrate transient productive HPV infection and spontaneous regression occurs in approximately 60% of cases under monitoring, while progression to CIN2 + occurs in only 11% and to cervical cancer in less than 0.5% of patients [2]. These favorable natural history data underpin current international guidelines that endorse active surveillance instead of immediate intervention for CIN1. Nevertheless, long-term follow-up data challenge the notion that the risk of progression for low grade cervical intraepithelial lesions is uniformly low. A nationwide, registry-based cohort study [3] demonstrated that the five-year cumulative incidence of CIN2+ among women with histologically proven CIN1 reaches 19%, rising to 25–26% in women with high-grade cytology or HPV16/18-positive status. These findings highlight a clinically significant subset of CIN1 patients at substantial risk of disease progression and underscore the need for interventions capable of enhancing the spontaneous regression rate of the wait-and-see approach alone.

The immunological environment of the cervical epithelium is a major determinant of whether HPV infection persists or is cleared, with effective viral clearance depending on a robust cell-mediated Th1-type immune response [4]. Plant sterols and sterolins, phytochemicals of which beta-sitosterol (BSS) and its glucoside (BSSG) are the principal bioactive constituents, have been shown to specifically modulate T-helper lymphocyte activity, selectively enhancing Th1-associated cytokines (interleukin-2, interferon-gamma) while suppressing the Th2-associated cytokine interleukin-4, thereby restoring immunological balance in conditions of chronic viral infection [5]. Flow cytometric studies in HIV-infected patients confirmed that administration of the BSS-BSSG mixture shifted a pathological Th2-predominant response toward a protective Th1 profile, mirroring the pattern observed in healthy controls [6]. Clinically, this immunomodulatory mechanism has been previously tested in the management of HPV-associated anogenital warts, according to the results of a randomized trial demonstrated that BSS-BSSG supplementation (Moducare) significantly improved the clearance of anogenital warts when combined with cryotherapy. It was found that lesion-free rates of 79.4% were observed at combination arm (cryotherapy plus six month Moducare) versus 61.7% at cryotherapy only arm [7].

Based on the fact that the underlying mechanism of promoting the cellular immunity against persistent HPV is directly relevant to the pathogenesis of CIN1, there is a compelling biological rationale to evaluate Moducare for this indication. Therefore, the MODUCIN-1 trial is designed to test the hypothesis that adjunctive immunomodulatory treatment can meaningfully increase and robust the rate of histological regression of CIN1 compared with surveillance alone. It is a prospective, open-label, randomized controlled trial that will allocate 182 women with histologically proven CIN1 in a 1:1 ratio to either a Wait-and-See group (control arm) or to six-month oral Moducare supplementation group (intervention arm), with the primary endpoint of CIN1 regression at six months.

## Materials and methods

### Trial design

**Fig 1:**
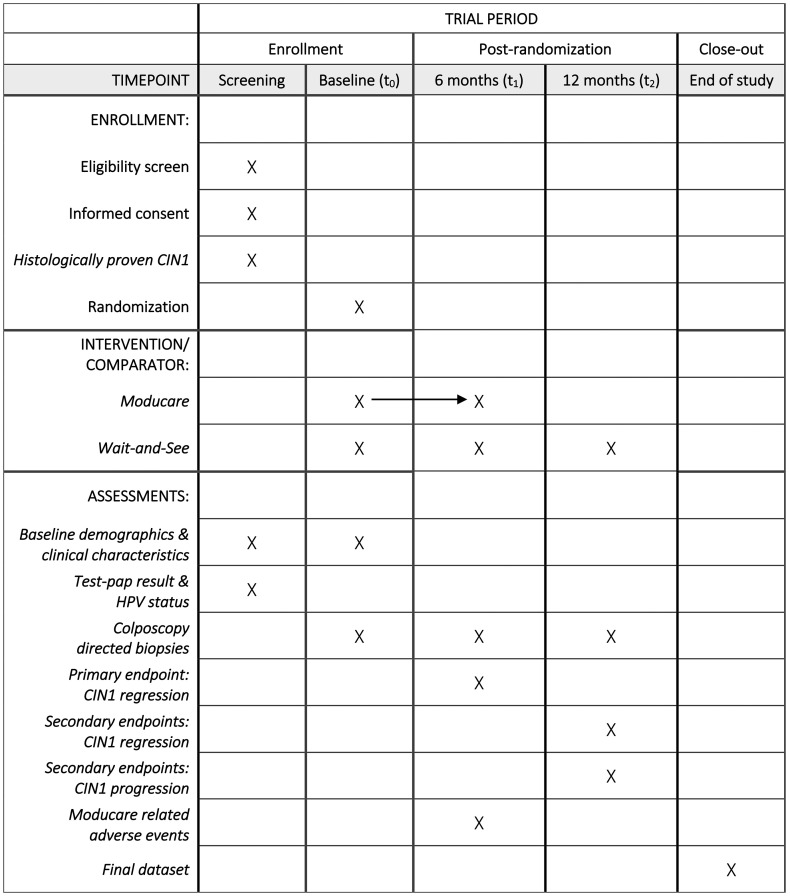
MODUCIN1 participant timeline (SPIRIT Schedule).

**Fig 2:**
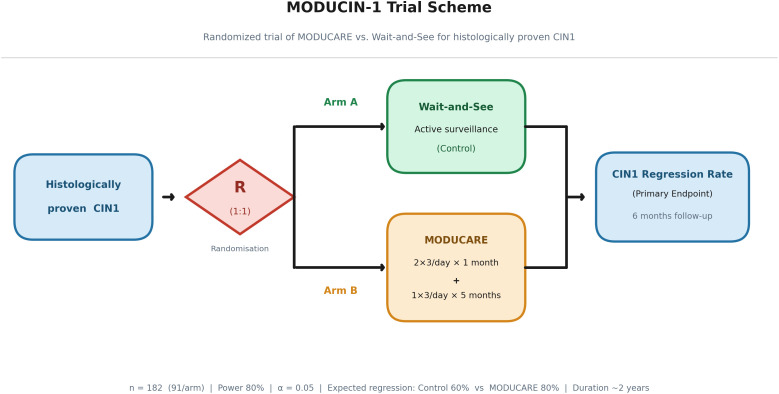
MODUCIN1 trial scheme.

This is a prospective, open-label, randomized trial, with no patient or public involvement. Patients are eligible for enrollment irrespective of any HPV status and Pap test result. Participants will be included in the study with histologically proven CIN1, after colposcopy-directed cervical biopsies. During colposcopy, the cervix is divided into four quartiles and based on the location of suspicious acetowhite areas, one biopsy per quartile is obtained. Patients will undergo randomization into the following two groups: Wait-and-see approach with active monitoring, designed as control arm A, or six months treatment with oral capsules of Moducare, designed as intervention arm B. The participant timeline and trial scheme are presented in Fig 1 and Fig 2, respectively.

After randomization, patients in both groups will undergo follow-up for at least one year, with two scheduled visits at six and twelve months. At these time points participants will undergo colposcopy-directed cervical biopsies from the cervical quartiles that were predefined as suspicious from the first colposcopy at randomization. If a suspicious acetowhite area at another previous normal appearing quartile is identified a colposcopy-directed cervical biopsy would be also obtained. Similarly to the first colposcopy, one biopsy per quartile will be taken.

The monitoring/treatment plan for each arm is in detail described below.

- Control arm A: Patients in the wait-and-see approach will undergo active monitoring with colposcopy-directed biopsies at six and twelve months after randomization.
- Intervention arm B: Patients in the Moducare group will undergo a six-month treatment with Moducare oral capsules. The predefined dosage will be two capsules, three times per day for the first month, followed by one capsule, three times per day for the remaining five months. Moducare is classified as a natural dietary supplement, designed as an immune support supplement containing a patented blend of plant sterols and sterolins, primarily beta-sitosterol and beta-sitosterol glucoside, derived from plant sources.

Endocervical curettage (ECC) is optional together with the colposcopy-directed cervical biopsies, during colposcopy at all three time points (randomization, six- and twelve-month visits) in cases with transformation zone type III. During follow-up visits pap test and HPV-DNA or RNA tests are allowed according to national and international guidelines, at the attending physician’s discretion. In case of modification in the treatment plan in either arm (Moducare dose change, participant request or disease progression to CIN3), the patient will be dropped out of the trial. The setting of the trial is a tertiary, academic center, certified by ESGO for Gynecological Oncology training, with a fully equipped Colposcopy Unit and adequate workload in order to reach the needed sample size. All patients will be treated by three gynecological oncologist, certified in colposcopy from the Hellenic Society for Colposcopy and Cervical Pathology (HSCCP) and all results will be centrally reviewed by the director of the Colposcopy Unit, who will monitor data collection (at six months after randomization), perform an interim analyses and could decide to terminate the trial. The trial has been approved by the local Scientific Committee and it is registered on clinicaltrials.gov (NCT07379905).

### Study population

It is estimated that the total number of participants that will be enrolled in the trial and randomized to the two arms is 182. The major inclusion criterion is histologically confirmed only low-grade cervical intraepithelial neoplasia (CIN1), with any HPV status and test pap results, while the major exclusion criterion is inadequate colposcopy due to vaginal stenosis or other anatomical reasons with no visible suspicious acetowhite lesions on the cervix and CIN1 result from the endocervical curettage (ECC) in cases with transformation zone type III. All patients will be assessed for the inclusion and exclusion criteria of MODUCIN1 trial. Those criteria are in detail described in Table 1.

**Table 1:** Inclusion and Exclusion criteria.

**Inclusion criteria**	Age 18–85 years old
	ECOG Performance status 0–1
	Histologically proven low-grade cervical intraepithelial neoplasia (CIN1)
	Any Pap test result
	Any HPV status (negative, positive: high or low risk)
**Exclusion criteria**	Pregnancy
	Low likelihood of patient compliance to treatment protocol and follow-up
	Inadequate colposcopy
	Previous operation to the cervix
	Previous pelvic malignancy
	Previous history of histologically proven CIN1, at least before 12 months.
	Previous histologically proven with or without treatment high-grade cervical intraepithelial neoplasia (CIN2, CIN3)
	Hypersensitivity to trial medication

### Trial endpoints

The primary endpoint of the trial is to compare the regression rate of low-grade cervical intraepithelial neoplasia (CIN1), between the two arms: Wait-and-see control arm and Moducare intervention arm, at six months after randomization. Furthermore, the secondary endpoints include the comparison of regression rate of CIN1 at twelve months from randomization, the progression rate of CIN1 at six and twelve months after randomization and for safety analysis the intervention (Moducare) related adverse events at six months after randomization. All comparisons will be made between the above-mentioned arms of the trial.

### Sample size

For power analysis of the trial a two-sided alpha level of 0.05, a statistical power of 80% and accounted for a 10% drop-out rate. Based on the results of a large recent meta-analysis [2] the spontaneous regression rate of low-grade cervical intraepithelial neoplasia (CIN1) of the Wait-and-see approach was set at 60% and in order to achieve a clinically important increase of at least 20%, the regression rate of Moducare was set at 80%, since robust RCTs are absent. The sample size was calculated using RStudio (V 4.3.3), applying the chi-square test formula for comparison of two independent proportions. Accordingly, we plan to enroll approximately 182 patients to account for anticipated dropouts and maintain the integrity of the primary endpoint analysis.

### Randomization

The primary block randomization with a 1:1 allocation ratio and a fixed block size of 2 will be used to assign each participant either to control arm A (Wai-and-see) or intervention arm B (Moducare). The randomization list will be generated in R (version 4.5.1) using the randomizeR package [8]. After confirming that a candidate participant fulfils all eligibility criteria, the investigator will access the randomization system to obtain the next available randomization code and the corresponding preassigned study arm for that participant.

### Study timeline

Patient enrollment is expected to start in the second quarter (Q_2_) of 2026 (01/06/2026). Before enrollment all patients will sign a written informed consent. Trial duration is estimated at twenty-four months, while participant recruitment is expected to last for twelve months [approximately the second quarter of 2027 (31/05/2027)]. The primary endpoint, regression rate of low-grade cervical intraepithelial neoplasia (CIN1), is expected to be reached after six months from last patient enrollment. Moreover, for the secondary endpoints: regression rate of CIN1 is expected to be reached after twelve months from last patient enrollment, while progression rate of CIN1 will be evaluated at two timepoints, after six and twelve months from last patient enrollment. The safety analysis include the investigation of the six-month intervention (Moducare) related adverse events, which will be assessed at six months from last patient enrollment of arm B. In conclusion, data collection is set to be completed in the second quarter of 2028 (31/05/2028), while the preliminary results are expected at the end of 2027 (01/12/2027) with the final results at the second quarter of 2028 (31/06/2028).

### Statistical methods

The primary analysis will follow the intention‑to‑treat (ITT) principle, including all randomized participants according to their allocated arm regardless of missing data or protocol deviations. A per‑protocol (PP) analysis will be performed as a sensitivity analysis, restricted to participants who completed the assigned intervention without major protocol violations. Baseline demographic and clinical characteristics will be summarized per arm using descriptive statistics. Means with standard deviations or medians with interquartile ranges for continuous variables, and frequencies with percentages for categorical variables. The balance between arms will be assessed descriptively.

Furthermore, the primary endpoint, regression rate of CIN1 at six months, will be compared between the two arms using the chi‑square test or Fisher’s exact test, as appropriate based on expected cell counts. An absolute risk difference with the corresponding 95% confidence interval will be reported as the primary effect measure. Additionally, the relative risk and odds ratio with 95% confidence intervals will be estimated. The same analytical approach will be applied to the secondary endpoints: regression rate at twelve months and progression rates at six and twelve months. Univariable and multivariable logistic regression models will be used to estimate odds ratios and 95% confidence intervals for regression and progression of CIN1. The multivariable model will include prespecified covariates and the model goodness‑of‑fit will be evaluated using the Hosmer‑Lemeshow test.

Furthermore, adverse events in the Moducare arm will be assessed by type and severity using descriptive statistics. Between‑arm comparison of the overall adverse event rate will be performed using Fisher’s exact test. In addition, subgroup analyses will be conducted to explore potential treatment‑effect modification: e.g., HPV status (HPV 16/18 positive vs. high-risk HPV vs. low-risk HPV) and smoking status (smoker vs. non‑smoker). These analyses are exploratory and will be interpreted with caution given the limited sample size. All statistical tests will be two‑sided with a significance level of p-value < 0.05. Statistical analyses will be performed using R (version 4.5.1; R Foundation for Statistical Computing, Vienna, Austria). All data will be stored on secure institutional servers with regular backup, and exports to statistical software will be performed via controlled, documented procedures.

### Participant retention

To minimize dropouts during the 12-month follow-up, we will implement a structured retention strategy coordinated by the primary investigator. Flexible scheduling, provision of appointment confirmations and results summaries will be offered. Contact details (phone and email) will be collected at baseline and confirmation reminders will be sent 7 days and 48 hours before each scheduled visit by SMS and email; non-responders will receive a telephone call within 7 days of a missed appointment. Participants will receive a printed schedule, a clinic contact card, and a brief leaflet explaining the importance of follow-up for their care and for study validity. Moreover, the primary investigator will register the reasons for withdrawal or missed visits, while the drop-out rate will be monitored monthly and an interim evaluation of retention will occur at enrollment midpoint, with corrective actions (extra reminders, outreach calls) if attrition exceeds the planned 10% allowance.

### Data management and Quality control

All study data will be entered into a secure, password-protected electronic database on institutional servers. Source documents (colposcopy notes, pathology reports, case report forms) will be retained in the trial file. Data entry will be performed by the three co-investigators of the study, following a coded patient ID system to preserve confidentiality. Regular weekly encrypted backups will be retained according to institutional policy. Furthermore, data quality control will include a 10% of random cross-checks against source documents during the first 50 enrollments and thereafter regular spot-checks and automated validation rules in the database to flag missing or out-of-range values. Adverse events, protocol deviations, and biopsy/pathology discrepancies will be reviewed at monthly trial meetings, while central pathology review will resolve discordant histology. The final dataset for analysis will be locked after resolution of queries and a documented audit trail will be retained.

## Discussion

The MODUCIN1 trial is designed to evaluate whether Moducare, a plant sterol and sterolin supplement containing beta-sitosterol (BSS) and its glucoside (BSSG), can enhance the spontaneous regression of histologically confirmed low-grade cervical intraepithelial neoplasia (CIN1) compared with the standard wait-and-see approach. Based on the previous reported immunomodulatory properties of the BSS-BSSG mixture and prior clinical evidence demonstrating its efficacy in promoting clearance of HPV-associated anogenital warts [7], we anticipate that the Moducare group will demonstrate a significantly higher regression rate at six months compared to the control arm. Specifically, we hypothesize that the intervention will increase the CIN1 regression rate from the expected 60% under surveillance alone [2] to approximately 80%, representing a clinically meaningful absolute difference of 20 percentage points. If confirmed, these results would also be expected to translate into lower progression rates at both six and twelve months in the intervention arm.

The biological plausibility of this hypothesis is supported by the known mechanism of action of BSS-BSSG, which selectively enhances Th1-associated cytokines (interleukin-2, interferon-gamma) while suppressing the Th2-associated cytokine interleukin-4 [5], thereby restoring the immunological balance required for effective HPV clearance [4]. Furthermore, in vitro evidence has demonstrated that beta-sitosterol can suppress HPV E6 expression and upregulate p53 in cervical carcinoma cell lines, suggesting a direct antiviral and tumor-suppressive effect beyond immunomodulation alone [9]. Additionally, the favorable safety profile of Moducare as a natural dietary supplement justified it as a beneficial adjunct to standard surveillance.

Should the trial confirm our hypothesis, the implications for clinical practice would be substantial. Currently, the management of CIN1 relies exclusively on passive monitoring, which, despite its favorable overall natural history, leaves a clinically significant subset of patients at risk of progression, particularly those with HPV16/18-positive status, where the five-year cumulative incidence of CIN2 + can reach 25–26% [3]. The incorporation of an immunomodulatory supplement into the surveillance strategy could represent a paradigm shift in the management of low-grade cervical dysplasia, offering a non-invasive, well-tolerated intervention that actively promotes lesion regression. This approach could reduce the psychological burden associated with prolonged surveillance and decrease the need for subsequent excisional procedures.

On the other hand, some limitations of the MODUCIN1 trial should be acknowledged. The open-label design introduces the potential for performance and detection bias, as neither participants nor investigators are blinded to group allocation. While a placebo-controlled design would have been methodologically preferable, it was not feasible in the context of a dietary supplement trial where a truly inert placebo matching the appearance, taste, and dosing regimen of Moducare capsules was not available. Nevertheless, the use of an objective histological endpoint, assessed by colposcopy-directed biopsies that were centrally reviewed by the director of the Colposcopy Unit, mitigates the impact of observer bias on the primary outcome. Furthermore, the fact that pathological assessment of biopsy specimens is inherently blinded to treatment allocation provides an additional layer of objectivity.

Moreover, the six-month primary endpoint, while clinically practical and aligned with standard follow-up intervals for CIN1, may not capture the full trajectory of treatment effect, as some CIN1 lesions require longer periods for regression. The twelve-month secondary endpoint assessment partially addresses this limitation. Finally, adherence to a six-month oral supplement regimen is inherently challenging in dietary supplement trials [10], and the absence of an objective biomarker to confirm supplement intake (such as serum beta-sitosterol levels) means that treatment adherence will rely on patient self-report and capsule counting.

The dissemination plan of the study include the publication of the final results of the study in international peer-review scientific journals. Preliminary and final results may also be presented in national and international seminars and congresses relevant to colposcopy and cervical pathology.

### Statement of ethics

This clinical trial protocol was approved by the Institutional Review Board of “Papageorgiou” General Hospital (no. 2025-Β2015–518, date: 14/01/2026). For any protocol amendment a new approval will be obtained Institutional Review Board. The study will be conducted according to the Declaration of Helsinki and all potential study participants will be given a written informed consent before enrolment. Patients’ data will be available upon request from the corresponding author for the reproducibility of this study.

## Supporting information

S1 File SPIRIT Checklist. (DOCX)

S1 File Study Protocol approved by Ethics Committee. (PDF)
