# Supplementary material for: Comparison of Moducare versus Wait-and-See approach for histologically proven Low-grade Cervical Intraepithelial Neoplasia (CIN1) (MODUCIN1 TRIAL) – Study protocol
Source: PLoS One. 2026 Jul 13;21(7):e0353119. doi: 10.1371/journal.pone.0353119 (PMC13362139; doi:10.1371/journal.pone.0353119)
Supplement: S1 File — (PDF) [file pone.0353119.s002.pdf]

MODUCIN1 TRIAL

(MODUcare for CIN1)

Τυχαιοποιημένη μελέτη MODUCARE vs. Παρακολούθησης για ιστολογικά επιβεβαιωμένη χαμηλού βαθμού τραχηλική ενδοεπιθηλιακή νεοπλασία (CIN1).

Γενικές Πληροφορίες

- Χορηγός: ΙΝΠΑ
- Κύριοι Ερευνητές: Δημήτριος Τσολακίδης, Δημήτριος Ζουζούλας
- Συνεργαζόμενοι Ερευνητές: Κίμων Χατζησταματίου
- Κέντρο Μελέτης: Α’ Μαιευτική - Γυναικολογική Κλινική ΑΠΘ, Γ.Ν. «Παπαγεωργίου»
- Αριθμός ClinicalTrials.gov: NCT07379905

Σύνοψη Μελέτης

Τίτλος

Τυχαιοποιημένη μελέτη MODUCARE vs. Παρακολούθησης για ιστολογικά επιβεβαιωμένη χαμηλού βαθμού τραχηλική ενδοεπιθηλιακή νεοπλασία (CIN1).

Ακρωνύμιο

MODUCIN-1 (MODUcare for CIN1)

Σχεδιασμός Μελέτης

Προοπτική, μονοκεντρική, ανοιχτού τύπου, τυχαιοποιημένη κλινική μελέτη

Πρωτεύον Αποτέλεσμα

Ποσοστό υποστροφής του CIN1 (Χρονικό πλαίσιο: 6 μήνες μετά την τυχαιοποίηση)

Δευτερεύοντα Αποτελέσματα

- Ποσοστό υποστροφής του CIN1 (Χρονικό πλαίσιο: 12 μήνες μετά την τυχαιοποίηση)
- Ποσοστό εξέλιξης του CIN1 (Χρονικό πλαίσιο: 6 & 12 μήνες μετά την τυχαιοποίηση)
- Ανεπιθύμητες ενέργειες σχετιζόμενες με τη θεραπεία (Χρονικό πλαίσιο: 6 μήνες μετά την τυχαιοποίηση)

Κριτήρια Εισαγωγής

- Ιστολογικά επιβεβαιωμένο CIN1
- Οποιοδήποτε HPV αποτέλεσμα (αρνητικό, θετικό: χαμηλού ή υψηλού κινδύνου)
- Οποιοδήποτε Pap test αποτέλεσμα
- Ηλικία 18 – 85 ετών
- ECOG: 0 – 1

Κριτήρια Αποκλεισμού

- Εγκυμοσύνη
- Χαμηλή πιθανότητα συμμόρφωσης της ασθενούς στο πρωτόκολλο θεραπείας και παρακολούθησης
- Προηγούμενη επέμβαση στον τράχηλο
- Προηγούμενη κακοήθεια στην πύελο
- Προϋπάρχον ιστολογικά επιβεβαιωμένο CIN1 (>12 μήνες).
- Προϋπάρχον ιστολογικά επιβεβαιωμένο CIN2 ή/και CIN3.
- Υπερευαισθησία στο υπό δοκιμή φαρμακευτικό σκεύασμα.

Τυχαιοποίηση

- Θα εφαρμοστεί μέθοδος τυχαιοποίησης κατά ομάδες (block randomization) με λόγο κατανομής 1:1 και μέγεθος ομάδας (block size) ίσο με 2, για την κατανομή κάθε συμμετέχοντα είτε στην Ομάδα Α (Παρακολούθηση) είτε στην Ομάδα Β (Moducare). Η λίστα τυχαιοποίησης θα παραχθεί με τη χρήση του λογισμικού R (έκδοση 4.5.1) και του πακέτου randomizeR. Μετά την επιβεβαίωση ότι κάθε υποψήφιος συμμετέχων πληροί τα κριτήρια εισαγωγής στη μελέτη, ο ερευνητής θα αποκτά πρόσβαση στο σύστημα τυχαιοποίησης, προκειμένου να ανακτήσει τον αντίστοιχο τυχαίο αριθμό και τον προκαθορισμένο βραχίονα στον οποίο θα ενταχθεί ο συμμετέχων

Θεραπεία

- Ομάδα Α: Ομάδα ελέγχου → Παρακολούθηση

- Ομάδα Β: Ομάδα θεραπείας → MODUCARE* κάψουλες από του στόματος., δοσολογία 1 x 3 για 6 μήνες (σύμφωνα με τις οδηγίες του κατασκευαστή).

* Το MODUCARE ταξινομείται ως φυσικό συμπλήρωμα διατροφής, ειδικά ως ανοσορυθμιστικό, που περιέχει πατενταρισμένο μείγμα φυτικών στερολών και στερολινών (κυρίως βήτα-σιτοστερόλη και βήτα-σιτοστερόλη γλυκοζίτη) από φυτικές πηγές. Από του στόματος χορήγησης, 1 κάψουλα 3 φορές ημερησίως για 6 μήνες.

Περιγραφή Μελέτης

Ο ανθρώπινος ιός των κονδυλωμάτων (HPV) σχετίζεται αιτιολογικά με τον καρκίνο του τραχήλου της μήτρας και τις προκαρκινικές δυσπλασίες. Οι δυσπλασίες εντοπίζονται κυρίως στη ζώνη μετάπλασης και διαγιγνώσκονται με κολποσκόπηση και βιοψία. Στις υψηλόβαθμες αλλοιώσεις η αντιμετώπιση περιλαμβάνει την κωνοειδής εκτομή, ενώ στις χαμηλόβαθμες προτιμάται η παρακολούθηση. Ωστόσο, η προσέγγιση αυτή μπορεί να βελτιωθεί μέσω συμπληρωματικών παρεμβάσεων, όπως με το MODUCARE.

Η μελέτη προβλέπει τυχαιοποίηση **182 ασθενών** (λόγος 1:1 μεταξύ των ομάδων). Το μέγεθος του δείγματος εκτιμήθηκε με βάση τα αναμενόμενα ποσοστά υποστροφής του CIN1 (παρακολούθηση: 60% και MODUCARE: 80%), με δύναμη 80% και επίπεδο σημαντικότητας 0.05 και ποσοστό αποχώρησης 10%.

Διάρκεια/Σχεδιασμός Μελέτης

Η συνολική διάρκεια της μελέτης εκτιμάται στα 2 έτη. Η ένταξη ασθενών στην μελέτη προβλέπεται να ολοκληρωθεί σε 1 έτος, ενώ το πρωτεύον και τα δευτερεύοντα αποτελέσματα αναμένονται στα 2 έτη μετά την τυχαιοποίηση.

MODUCIN1 TRIAL

(MODUcare for CIN1)

A randomized trial of MODUCARE versus Wait-and-See Approach for histologically proven low-grade Cervical Intraepithelial Neoplasia (CIN1).

General Information

- Sponsor: INPA
- Principal Investigators: Dimitrios Tsolakidis, Dimitrios Zouzoulas
- Sub-Investigators: Kimon Chatzistamatiou
- Trial Site: 1st Department of Obstetrics and Gynecology AUTh
- ClinicalTrials.gov number: NCT07379905

Trial Synopsis

Title

A randomized trial of MODUCARE versus Wait-and-See Approach for histologically proven low-grade Cervical Intraepithelial Neoplasia (CIN1).

Acronym

MODUCIN-1 (MODUcare for CIN1)

Trial design

Prospective, single center, open-label, randomized trial

Primary endpoint

Regression rate of CIN1 (Time frame: 6 months after randomization)

Secondary endpoints

- Regression rate of CIN1 (Time frame: 12 months after randomization)
- Progression rate of CIN1 (Time frame: 6 & 12 months after randomization)
- Treatment related adverse events (Time frame: 6 months after randomization)

Inclusion criteria

- Histologically proven CIN1
- Any HPV status (negative, positive: high or low risk)
- Any Pap test result
- Age 18 – 85 years old
- ECOG Performance status 0 – 1

Exclusion criteria

- Pregnancy
- Low likelihood of patient compliance to treatment protocol and follow-up
- Previous operation to the cervix
- Previous pelvic malignancy
- Pre-existing histologically proven CIN1 > 12 months
- Pre-existing histologically proven CIN2 and/or CIN3
- Hypersensitivity to trial medication

Randomization

- 1:1 randomization into a control arm (wait-and-see) and a treatment arm (Moducare)

Therapy

- ARM A: Control Arm → Wait-and-See approach

- ARM B: Intervation Arm → MODUCARE* oral capsules., dosage 2 x 3 for 1 month and 1 x 3 for 5 months (total 6 months, as per instructions provided by the manufacturer)

* MODUCARE is classified as a natural dietary supplement, specifically an immune support supplement containing a patented blend of plant sterols and sterolins, primarily beta-sitosterol and beta-sitosterol glucoside, derived from plant sources. It is taken orally, 1 capsule three times daily for 6 months.

Trial description

Human Papillomavirus (HPV) is causally associated with cervical cancer and precancerous lesions (dysplasias) of the cervix. These lesions are detected mainly in the transformation zone and are diagnosed with colposcopy and biopsy confirmation. In high-grade lesions conization (surgical removal of a cone tissue form the cervix) is the therapy of choice, but in low-grade lesions monitoring and no-treatment is preferred. However, this wait-and-see approach can be assisted by pharmaceutical treatment, like MODUCARE.

The plan is to randomize **182 patients** in 1:1 ratio into ARM A : ARM B. Sample size was estimated based on the expected regression rates in each group (Wait-and-see: 60%, MODUCARE: 80%), selecting statistical parameters (power 80%, alpha 0.05), using a standard formula for comparing two independent proportions and a 10% expected drop out or loss.

Trial duration/schedule

Trial duration is estimated at 2 years. Accrual is excepted to last for 1 year and the primary and secondary endpoints are expected to be reached 2 years after randomization.
